# Impact of FDG-PET/CT for the Detection of Unknown Primary Tumours in Patients with Cervical Lymph Node Metastases

**DOI:** 10.4274/Mirt.344

**Published:** 2012-08-01

**Authors:** İnanç Karapolat, Kamil Kumanlıoğlu

**Affiliations:** 1 Şifa University School Medicine, Deparment of Nuclear Medicine, İzmir, Turkey; 2 Ege University School Medicine, Deparment of Nuclear Medicine, İzmir, Turkey

**Keywords:** Neoplasms, unknown primary, lymphatic metastasis, neoplasm metastasis, Fluorodeoxyglucose F18, positron-emission tomography/computed tomography, head and neck neoplasms

## Abstract

**Objective:** Because the detection of the primary tumour is of importance to optimize the patient’s management and allows a targeted therapy, the performance of hybrid positron emission tomography–computed tomography (PET/CT) using fluorodeoxyglucose (FDG) in the detection of primary tumors and unrecognized metastases with cervical lymph node metastases were evaluated in a retrospective study.

**Material and Methods:** Twenty patients with cervical lymph node metastases of unknown primary tumors underwent staging with FDG-PET/CT. All underwent head and neck examinations, computed tomography (CT), and/or magnetic resonance imaging (MRI), panendoscopies, and biopsies of head and neck mucosal sites. The diagnostic accuracy of FDG-PET/CT in detecting primary tumors was compared with that of histopathology and clinical follow-up. The ability of FDG-PET/CT to detect distant metastases was also tested.

**Results:** PET/CT was positive with an increased FDG uptake suggesting the potential primary site in 45% of patients (9/20). PET/CT findings were true positive in 7, true negative in 10, false positive in 2, and false negative in 1 patients, resulting in a sensitivity of 87%, a specificity of 83%, an accuracy of 85%, a positive predictive value of 77% and a negative predictive value of 90%. Also, PET/CT showed distant metastases in seven patients.

**Conclusion:** FDG-PET/CT can be successfully used for the identification of the primary site and distant metastases in patients with cervical lymph node metastases from an unknown primary cancer.

**Conflict of interest:**None declared.

## INTRODUCTION

Carcinomas of unknown primary are the seventh most frequent type of cancer in the world (1) In about 2-10% of all newly diagnosed biopsy-confirmed malignancies, the site of origin is not identified by routine clinical workup and they are thus categorized as carcinoma of unknown primary cancer. In 24-36% of these patients, the prior localization of the first carcinoma of unknown primary cancer manifestation are lymph nodes in the head and neck region (2). Both the location and the cytological typing of the metastatic cervical lymph nodes may give an indication as to the location of the primary tumour. The most common cytopathological findings in metastatic cervical lymph nodes originating from an unknown primary tumour are squamous cell carcinoma (SCC), undifferentiated carcinoma and adenocarcinoma. If the metastatic lymph nodes are located in the upper and middle cervical levels, especially in the case of SCC, a primary tumour in the head and neck region is likely. If only the lower cervical lymph nodes are involved, the primary tumour is often located below the clavicles. Also, cervical metastases of non-SCC and other solid tumours at any level can be due to a primary tumour outside the head and neck area (3). Unknown primary tumour prognosis is poor, the median survival in unknown primary tumour patients enrolled in clinical studies is as short as 6-10 months. However, the outcome is more favourable in patients presenting with cervical unknown primary tumour, with a 5-year survival ranging from 35 to 50%. This is especially true when the primary tumour of a cervical unknown primary tumour is detected because it allows targeted therapy (radiation planning and/or decision of surgery) (4). The usual diagnostic work-up consists of a physical examination, fiber optic laryngoscopy and nasopharyngoscopy, conventional imaging, i.e. computed tomography (CT) and/or magnetic resonance imaging (MRI), panendoscopy with biopsies directed on the suspected primary tumour sites or randomized in the most frequent sites of primary tumour, and sometimes tonsillectomy if the metastatic lymph nodes are located in the upper and middle cervical levels, especially in the case of SCC. Patients with metastatic adenocarcinoma the usual diagnostic work-up consists of a physical examination, gastrofiberscopy, colonoscopy, and chest/abdominal CT. In the case of negative morphological examinations (CT, MRI), F-18 fluorodeoxyglucose positron emission tomography/ computed tomography (FDG PET/CT) has been proposed as a useful tool to localize the primary tumour with extremely variable detection rates ranging from 8 to 53%. FDG PET has also been shown to detect unrecognized metastases in patients with cervical lymph node metastases from cancer of unknown primary. Because many neoplasms have higher glycolytic activity than normal tissue, primary and metastatic tumors show greater uptake of the glucose analog FDG, and appear as hot spots on PET images (5). The aim of this retrospective study was to evaluate the performances of FDG PET/CT in patients with cervical lymph nodes metastases from cancer of unknown primary concerning the detection of the primary tumour and/or distant lesions. 

## MATERIALS AND METHODS

Twenty patients (19 male, 1 female, mean age 55 years, range 20–76 years), with histologically or cytologically proven cervical lymph node metastases who underwent whole body dual modality FDG PET/CT imaging from March 2008 to May 2011 were included. Image evaluation was performed retrospectively. The study protocol was reviewed and approved by the Institutional Review Board of our hospital, and written informed consent was obtained from each patient.

Patients with a previous history of malignancies were excluded and none had received chemotherapy and/or radiation therapy prior to hybrid FDG PET/CT scan. Metastatic lymph node cytology revealed squamous cell carcinoma in 16 patients, adenocarcinoma in 1 patient and undifferentiated carcinoma in 3 patients. All patients were evaluated by careful physical and endoscopic examinations of the upper aerodigestive tract. After this routine diagnostic work-up, fifteen patients underwent head and neck CT and/or MRI and one patient with metastatic adenocarcinoma underwent gastrofiberscopy, colonoscopy and head and neck/chest/abdominal CT. When this work-up is completed, a hybrid FDG PET/CT scan is performed. Also four patients referred firstly to FDG PET/CT without conventional diagnostic work-up (Table 1).

**Imaging and Interpretation of Data **

Combined FDG PET/CT was performed using a Siemens HI-REZ biograph 6 which provides an in-plane spatial resolution of 4.8 mm, an axial field view of 16.2 cm, and three-dimensional image acquisition. Patients were required to fast for 6 hour prior to scanning, and whole-body PET scanning from the skull base to the upper thighs was performed approximately 1 h after the intravenous injection of 555 MBq of F-18 FDG. Whole body CT scanning was performed in cranio-caudal direction. Intravenous contrast was not used during CT scanning. Immediately after, PET data were collected in craniocaudal direction with the arms down. FDG PET images were reconstructed using CT data for attenuation correction.

Two experienced nuclear medicine physicians reviewed blindly and independently the hybrid FDG PET/CT scans as positive or negative for a primary tumour site. PET/CT was considered positive when an increased FDG uptake indicative of a primary tumour was identified in the head and neck and/or other regions of body. The degree of suspicion of malignant involvement was based on qualitative visual interpretation of the images and no quantitative analysis such as the measurement of standardised uptake values was performed. Also distant metastases are noted. PET/CT results were correlated with the patient’s medical record concerning pathological results and follow-up. The end points were the ability of hybrid FDG PET/CT to detect the potentially primary tumour and/or distant lesions. 

**Figure 1:**
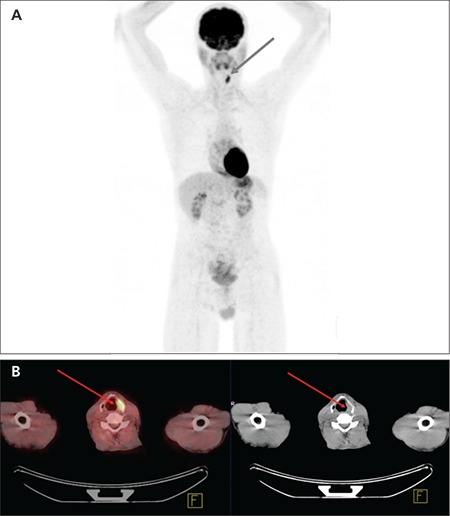
Detection of a primary tumour by combined FDG PET/CT. At the (A) MIP image and (B) PET/CT fusion and CT image of neck FDG PET/CT detected focal FDG uptake in the left larynx due to primary tumour that was later confirmed by histology

**Figure 2:**
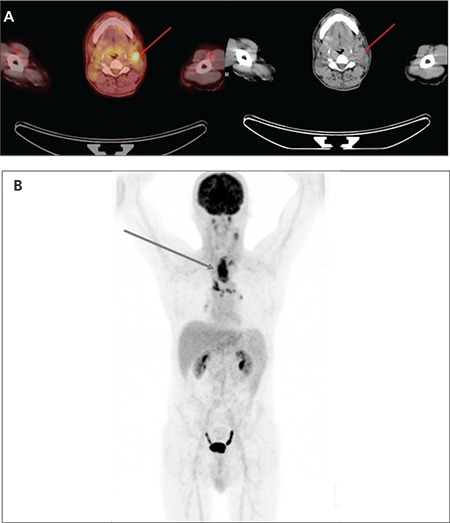
(A) PET/CT fusion and CT images of a patient referred for PET/CT due to cancer of unknown primary with a left cervical lymph node metastasis. (B) MIP image showed an esophageal tumour with additional mediastinal lymph node metastasis that was later confirmed by histology and follow-up

A PET/CT result was considered as a true positive when an FDG focus matched the primary tumour found during the second panendoscopy and biopsies (Figure 1, 2), a true negative when both FDG PET and second panendoscopy and biopsies did not find the primary tumour, a false positive when the increased FDG focal uptake did not match panendoscopy results and a false negative when the second panendoscopy and biopsies detected malignant lesions with no corresponding increased FDG focal uptake.

After the FDG PET/CT scan, the patients were re-assessed in a multidisciplinary approach, including a second panendoscopy and biopsies.

## RESULTS

FDG-PET/CT was found positive for a primary tumour in 9/20 patients (45%) and negative in 11/20 (55%) patients. PET/CT findings were true positive in 7, true negative in 10, false positive in 2, and false negative in 1 patients, resulting in a sensitivity of 87%, a specificity of 83%, an accuracy of 85%, a positive predictive value of 77% and a negative predictive value of 90%. Also PET/CT showed distant metastases in seven patients (Table 2).

## DISCUSSION

Several reports in the literature describe the efficacy of FDG-PET/CT in detecting unknown primary tumors in patients with cervical lymph node metastases (6). As detection of the primary has a great impact on both the curative loco-regional treatment and the prognosis of these patients, maximum effort should be made to localise the primary, even the search for a primary tumor is often costly and time-consuming. In the study by Haas et al., the 3-year survival rate in patients with identified head and neck primary tumours was 100% after treatment, compared to 58% in patients with unknown primary tumours (7). But as is known from the literature, there are still patients whose primary tumour will never be found (8). 

A meta-analysis of 16 studies found that FDG PET or PET/CT detected primary tumors in 74 of 302 (24.5%) cancer of unknown primary patients with neck node metastases, with an overall sensitivity, specificity, and accuracy of 88.3%, 74.9%, and 78.8%, respectively (6). In comparison, we found in our study that FDG PET/CT detected primary tumors in 7 of 20 (35%) cancer of unknown primary patients with neck node metastases, with an overall sensitivity, specificity, and accuracy in primary tumor detection of 87%, 83%, and 85%, respectively. We think that the relative higher rates of our study are due to small number of patients and using only PET/CT for imaging. Because it was shown that the diagnostic value in patients with head and neck cancer is higher with PET/CT when compared to both modalities taken separately, as PET/CT increased the overall correct staging. In the study by Gutzeit et al., PET/CT revealed the primary site of malignancy in 33% of patients with cervical metastases versus 22% for PET alone (9). 

In our study, sixteen FDG PET/CT examinations were performed after negative diagnostic work-up. In 10 of 16 patients FDG PET/CT couldn’t also find the primary tumor. In 6 of 16 patients FDG PET/CT was found positive for a primary tumour and after re-assessing in a multidisciplinary approach, including a second panendoscopy and biopsies, in 4 of 6 patients the primary tumor was confirmed as lung, tonsil, esophagus and the base of tongue cancer (number 5, 7, 12 and 20) respectively. But 2 patients (number 6 and 14) that FDG PET/CT was found positive for primary stomach and nasopharynx cancer, the second endoscopy and biopsies couldn’t confirm the diagnosis. We think that the reason of false positive results could be due to physiologic FDG uptake at nasopharynx and stomach that makes misinterpretation of study. Also physiological FDG uptake can be variable in the tonsils, adenoids, salivary glands and in muscles of the head and neck that renders image analysis difficult and may be the cause for false positive findings (6). The tonsils were the most common site of false-positive FDG uptake (39.3%, compared with 28.3% for all other sites combined). The high rate of false positive results and the low specificity of FDG-PET/CT for tonsillar tumors can be attributed to FDG uptake caused by increased cellular metabolism in inflammatory lesions. Li et al. found that inflammatory lesions had a mean FDG standardized uptake value (SUV) of 2.58 (standard deviation, 0.77) (10). Similarly, Adams et al. found that inflamed lymphoid tissue had SUV values that ranged from 2.0 to 15.8 (11). This enhanced FDG uptake by benign lesions in the tonsils overlaps with the range of uptake levels found in malignancies and can, therefore, lead to false-positive results. False-positive rates may be reduced by coregistration of PET and CT, thus providing both physiologic and anatomic imaging capabilities. The poor spatial resolution of PET has been overcome by fusing the anatomic results of CT with the functional results of PET (5).

In our study, there is one false negative patient (number 9), that FDG PET/CT couldn’t find the primary tumor, but after re-assessing there was a salivary gland tumor at the left parotis confirmed by surgery. We think that the reason of false negative result could be due to variable FDG uptake of salivary gland tumors. Other false negative results may be due to small primary tumours below the resolution of FDG PET (5 mm) or to a reduced signal-to-noise ratio caused by high background uptake and/or well differentiated tumors with relatively low FDG uptake. 

In our study, four FDG PET/CT examinations were performed initially before any diagnostic work-up. In 3 of 4 patients FDG PET/CT was found positive for the primary tumour (two larynx and one nasopharynx cancer) and afterwards panendoscopy and biopsy confirmed the diagnosis. 1 of 4 patients FDG PET/CT and other following diagnostic work-up couldn’t find the primary tumour. However, the exact position of FDG PET/CT in the diagnosis work-up of patients with cervical metastases from unknown primary tumour has probably to be reconsidered, it could be programmed as a first step in the diagnostic work-up. This would have the advantage of guiding biopsies to a potential primary site and ruling out any synchronous tumour. 

Hybrid FDG PET/CT also offers the opportunity to detect primary tumour below the clavicle, distant lesions and perform a complete staging in a ‘one step’ procedure. It is well known that a synchronous second tumour and/or metastases are frequently observed in this patient population. Detection of metastatic lesions is crucial to avoid a heavy surgical procedure. In our study, FDG-PET–CT detected distant metastases in seven patients and altered the treatment management. But there was no synchronous second tumour found with FDG PET/CT in our study. 

Main limitations of our study were the small number of patients and heterogeneity in patient pathology and diagnostic work-ups. But in conclusion we considered from this study that FDG PET/CT is a more sensitive, selective and decisive procedure for detecting unknown primary tumours with cervical metastases. In addition, FDG PET/CT of the whole body has the ability to detect unknown distant metastases and can alter the treatment management. Additionally, we therefore advocate FDG PET/CT as the first diagnostic modality in patients with a cervical metastasis of an unknown primary tumour when fine-needle aspiration biopsy has proven malignancy and physical examination cannot reveal the primary tumour, because the results of FDG-PET/CT screening can serve as a guide for biopsies and other imaging procedures if needed. It can thus probably play a key role in both the diagnostic work-up and the selection of curative or palliative treatment in any particular patient. But further studies are needed to determine its exact position in the diagnostic work-up and potential role in patient’s prognosis. 

## Figures and Tables

**Table 1:**
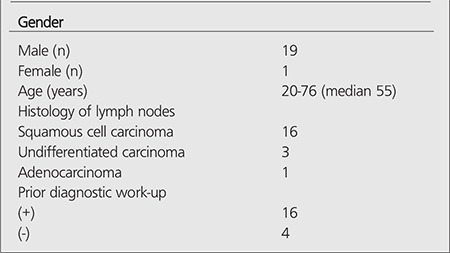
Patient and disease characteristics

**Table 2:**
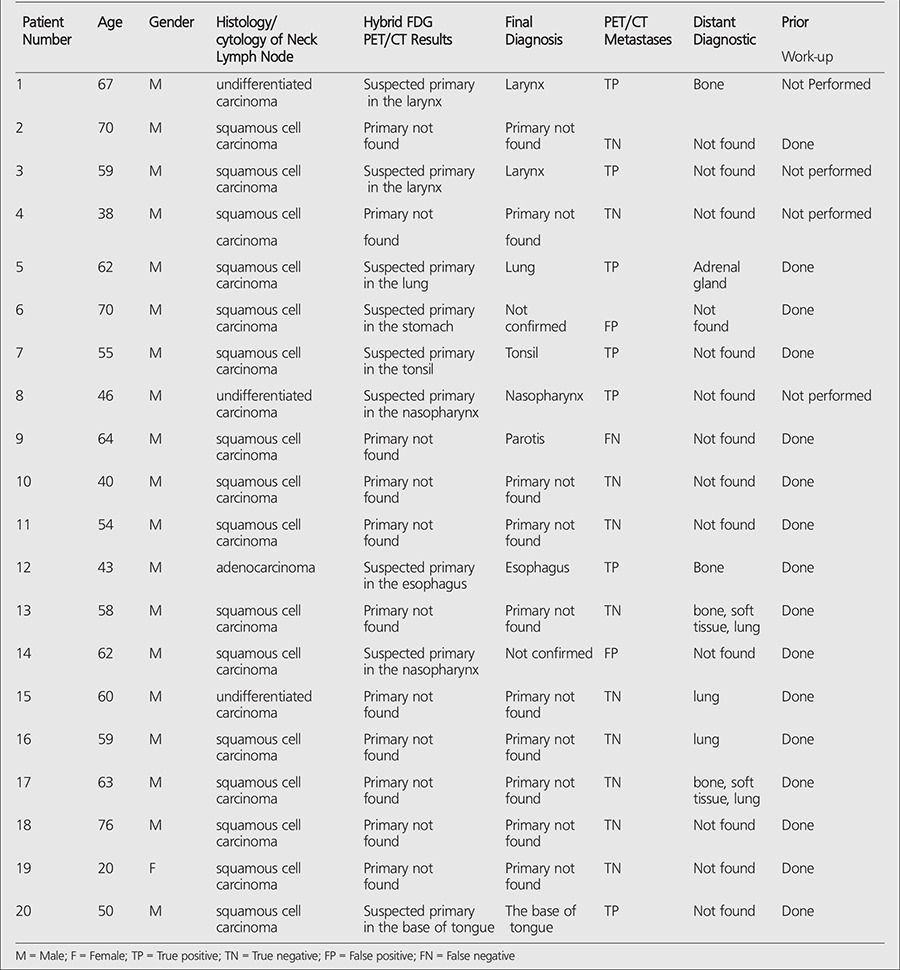
All data of the patients with cervical metastases of cancer of unknown primary
